# The G-protein-coupled formylpeptide receptor FPR confers a more invasive phenotype on human glioblastoma cells

**DOI:** 10.1038/sj.bjc.6605591

**Published:** 2010-03-02

**Authors:** J Huang, K Chen, J Chen, W Gong, N M Dunlop, O M Z Howard, Y Gao, X-w Bian, J M Wang

**Affiliations:** 1 Laboratory of Molecular Immunoregulation, Cancer and Inflammation Program, Center for Cancer Research, National Cancer Institute at Frederick, Frederick, MD 21702, USA; 2 Department of Pathophysiology, College of High Altitude Military Medicine, Third Military Medical University, Chongqing 400038, People's Republic of China; 3 Institute of Pathology and Southwest Cancer Center, Southwest Hospital, Third Military Medical University, Chongqing 400038, People's Republic of China; 4 Basic Research Program, SAIC-Frederick, National Cancer Institute at Frederick, Frederick, MD 21702, USA

**Keywords:** formylpeptide receptor, invasion, glioblastoma, cancer

## Abstract

**Background:**

The G-protein-coupled formylpeptide receptor (FPR) that mediates chemotaxis of phagocytic leucocytes induced by bacterial and host-derived chemotactic peptides is selectively expressed by highly malignant human gliomas and contributes to tumour growth and angiogenesis. As invasion of surrounding normal tissues is one of the important features of tumour malignancy, we investigated the function of FPR in the invasive behaviour of human glioblastoma cells.

**Methods:**

Cells (FPR^+^ and FPR^−^) were isolated by single-cell cloning from a human glioblastoma cell line U-87MG. The FPR expression was assayed by flow cytometry and reverse transcription PCR. The function of FPR was investigated by chemotaxis and calcium flux induced by FPR agonist fMLF. Tumour cell motility was assayed by a wound-healing model *in vitro*. The growth and invasive phenotype were observed with subcutaneous implantation of tumour cells in nude mice. Over-expression of FPR in FPR^−^ cells was performed by transfection of a plasmid vector-containing human FPR gene.

**Results:**

One of the glioma clones F9 that expressed high level of FPR showed a more ‘motile’ phenotype *in vitro* as compared with a clone G3 without FPR expression. Although F9 and G3 clones both formed subcutaneous tumours in nude mice, only F9 tumours invaded surrounding mouse connective tissues. Over-expression of FPR in G3 clone (G3F) increased the cell motility *in vitro* and the capacity of the cells to form more rapidly growing and invasive tumours in nude mice. We further found that, in addition to supernatant of necrotic tumour cells, foetal calf serum and human serum used in culture media contained FPR agonist activity and increased the motility of FPR-expressing glioblastoma cells.

**Conclusion:**

The expression of FPR is responsible for increased motility of human glioblastoma cells and their formation of highly invasive tumours.

Malignant gliomas are diffuse, often multifocal and invasive tumours with a dismal prognosis. A major barrier to effective treatment of malignant gliomas is their tendency to extend tendrils of tumour away from the main tumour mass. In fact, 95% gliomas recur within 2.5 cm of the resection margin, which contains residual tumour cells that act as a ‘disease reservoir’ (Giese *et al*, 2003). Malignant glioma cells acquire characteristic properties from a number of coordinated cellular programmes, including abberant expression of cell surface chemoattractant and growth-factor receptors, necessary for increased proliferation (e.g. expansion), migration (e.g. motility), invasion (e.g. extracellular matrix (ECM) degradation) (Demuth and Berens, 2004) and angiogenesis (e.g. production of angiogenic factors) (Giese *et al*, 2003).

Formylpeptide receptor (FPR) is a G-protein-coupled receptor (GPCR), originally identified in phagocytic leucocytes, that mediates cell chemotaxis and activation in response to the bacterial formylated chemotactic peptides (e.g. fMLF). Agonist binding to FPR elicits a signal transduction cascade involving phosphatidylinositol 3-kinase, protein kinase C (PKC), mitogen-activated protein kinases (MAPKs) and the transcription-factor nuclear-factor (NF)-*κ*B (Le *et al*, 2002; Cheng *et al*, 2007). Owing to its expression in cells of the immune system and its interaction with bacterial chemotactic peptides, FPR was thought to participate in host defence against microbial infection. In support of this notion, mice depleted of the FPR analogue mFPR1 were more susceptible to infection by *Listeria monocytogenes* (Gao *et al*, 1999) that produced high affinity mFPR1 agonist peptides (Southgate *et al*, 2008). Recently, a number of host-derived chemotactic agonists of FPR have been identified, including formylpeptides potentially released by mitochondria of ruptured cells (Rabiet *et al*, 2005), annexin I produced by activated epithelia (Xia *et al*, 2002) and a neutrophil granule protein, cathepsin G (Sun *et al*, 2004). In addition, functional FPR has been detected in cells of nonhaematopoietic origin, such as lung epithelial cells (Rescher *et al*, 2002) and hepatocytes (McCoy *et al*, 1995). These findings expanded the spectrum of pathophysiologic processes in which FPR may have a function.

As a support of the function for FPR in diseases other than anti-microbial host defence, our recent studies revealed that tumour cells of a majority of highly malignant human gliomas, including anaplastic astrocytoma (grade III) and glioblastoma multiforme (GBM, grade IV), express FPR (Zhou *et al*, 2005; Huang *et al*, 2008a). The FPR expressed in tumour cells derived from human GBM by responding to cognate agonist peptide fMLF and agonist(s) released by necrotic tumour cells promote the directional migration, survival and production of angiogenic factors by tumour cells (Zhou *et al*, 2005; Yao *et al*, 2008a). Depletion of FPR by short-interference RNA markedly reduced the tumourigenicity of GBM cells in immune-deficient mice (Zhou *et al*, 2005). Thus, both *in vitro* and *in vivo* evidence supports the potential for FPR to exacerbate the progression of highly malignant human glioma. As malignant tumours are composed of heterogeneous cells varying in their degree of differentiation or malignancy, and as increased motility and invasiveness define more highly malignant tumour cell subsets, we studied the contribution of FPR to the invasive capacity of human GBM cells. Here, we report that expression of FPR is sufficient to endow GBM cells with a more motile phenotype and with greater capacity to form tumours infiltrating surrounding normal tissues.

## Materials and methods

### Cells and reagents

Human GBM cell line U-87MG was obtained from the American Type Culture Collection (ATCC, Manassas, VA, USA). The cells were grown in Dulbecco's modified Eagle medium (DMEM) containing 10% foetal calf serum (FCS) and antibiotics; fMLF was purchased from Sigma-Aldrich (St Louis, MO, USA). Antibodies against phosphorylated Akt, Akt, phosphorylated p38, p38, phosphorylated Erk, Erk, phosphorylated EGFR (tyrosine 992) and EGFR were from Cell Signaling Technology (Beverly, MA, USA). Antibodies against vimentin, glial fibrillary acidic protein (GFAP) and FPR were from BD Biosciences (San Jose, CA, USA).

### Isolation of FPR-positive and -negative tumour cell clones

U-87MG cells were plated in 96-well plates at a concentration of a single cell per well. Wells containing either none or more than one cell were excluded for further analysis. After expansion of single-cell clones, the FPR^+^ and FPR^−^ subclones were identified by immunofluorescence staining of FPR, reverse transcription PCR (RT–PCR) and functional assays. One cell clone expressing high level of FPR was designated F9, whereas one clone, which was completely negative for FPR expression, was termed as G3.

### Over-expression of FPR in G3 cells

G3 cells were transfected with the Superfect Transfection Reagent (QIAGEN, Valencia, CA, USA) and 5 *μ*g FPR-expressing plasmid pcDNA3 or blank plasmid pcDNA3 (Invitrogen, Carlsbad, CA, USA). The FPR-transfected cells (G3F) and mock-transfected cells (G3M) were selected and maintained by incubation with 800 *μ*g ml^–1^ antibiotic G418. G3F cells were selected by flow cytometry sorting after staining with a PE-conjugated monoclonal antibody against FPR.

### Chemotaxis, Ca^2+^ flux and immunoblotting

Assays for tumour cell chemotaxis (Le *et al*, 2000; Zhou *et al*, 2005), Ca^2+^ mobilisation and immunoblotting were performed according to the described procedures (Le *et al*, 2000; Zhou *et al*, 2005).

### Wound-healing assays

The wound-healing assays were performed according to the published procedures (Liang *et al*, 2007). Briefly, tumour cells were labelled either by CellTracker Red CMPTX or CellTracker Green CMFDA (Invitrogen). Equal number of labelled cells were mixed and planted in 6 cm culture dishes. When the cells reached confluence, a 1000 *μ*l pipette tip (ThermoFisher Scientific, Waltham, MA, USA) was used to scratch and create a gap in the monolayer. Detached tumour cells were immediately removed by replacement of culture medium. The cell movement phase images were subsequently captured under light microscopy. The fluorescence time-lapse images were monitored by laser confocal microscopy. The results were quantified by calculating mean migrated distance of leading cells or the cell number filled in the scratched area (gate).

### Adhesion assays

Flat-bottomed 96-well plates were coated with 0.5% gelatin or 30 *μ*g ml^–1^ laminin at room temperature for 1 h followed by 1% heat-inactivated BSA for 30 min at room temperature. The plates were washed twice by PBS, and 100 *μ*l of 4 × 10^5^ per ml tumour cells were added to the wells. The plates were cultured for 10 min at 37°C and non-adherent cells were then removed by washing with ice-cold PBS. The number of adhering cells was evaluated by MTT method according to the kit manual (Sigma-Aldrich). The percentage of adhered cells in total input cells was calculated as the rate of adhesion.

### Zymography for MMP expression

Zymography (gelatin-substrate gel electrophoresis) was performed according to the described methods (Zhou *et al*, 2002) and the manual of the Zymography system (Invitrogen).

### Vascular endothelial growth factor production and microvascular density

Vascular endothelial growth factor (VEGF) in the supernatants of tumour cells was quantified by commercial enzyme-linked immunosorbent assay kits (Lymphokine Testing Laboratory, SAIC Frederick, Frederick, MD, USA). Frozen sections of xenograft tumours were stained with anti-human VEGF and PE-labelled second antibodies to visualise the VEGF expression, with anti-CD105 antibodies (Biolegend, San Diego, CA, USA) and immunochemical staining kit (LabVision, Fremont, CA, USA) to visualise the microvasculature.

### Flow cytometry, RT–PCR, immunocytochemistry tumourigenesis, histology, cell proliferation and tumour colony formation

Flow cytometry, RT–PCR, immunocytochemistry tumourigenesis, histology, cell proliferation and tumour colony formation were performed as reported earlier (Huang *et al*, 2007, 2008b).

### Statistical analyses

All experiments were performed at least three times and representative results are shown. A computer-aided Mann–Whitney *U*-test program SPSS (Version 11.0) (GraphPad Software Inc., San Diego, CA, USA) was used to determine the statistical significance of the differences between cell responses to testing materials and controls in experiments of chemotaxis, cell proliferation, adhesion, tumour colony formation and growth in nude mice.

## Results

### Isolation of FPR-positive and -negative subsets from U-87MG cells

**Figure 1:**
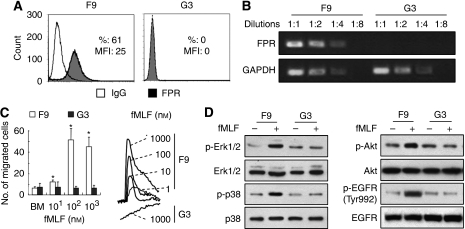
The FPR expression and function in FPR^+^ and FPR^−^ subclones from human U-87MG glioblastoma cells. Clones (FPR^+^ (F9) and FPR^−^ (G3)) were isolated by single-cell clone. (**A**) FACS analysis of FPR expression on tumour cells. The FPR expression was measured by a PE-conjugated mouse monoclonal antibody against FPR. %=percentage of FPR-positive cells; MFI=mean fluorescence intensity. (**B**) The expression of FPR mRNA. The levels of FPR mRNA were examined by RT–PCR. GAPDH was used as control. (**C**) The fMLF-induced chemotaxis and Ca^2+^ flux. Cell chemotaxis was assayed in response to fMLF. The results are expressed as the mean migrated cell number (±s.e.) in three high-powered fields of three independent experiments. ^*^Indicates significantly increased cell migration as compared with medium control (0) (*P*<0.01). The fMLF-induced Ca^2+^ flux was measured in a fluorescence spectrometer. The FPR agonist peptide fMLF-induced fluorescence intensity in F9 and G3 cells was expressed as the ratio at wavelength 340 out of 380 calculated by an FLWINLab program. (**D**) FPR-mediated activation of signal transduction molecules. Lysates of F9 (FPR^+^) and G3 (FPR^−^) cells stimulated with 100 nM fMLF for 10 min were examined for phosphorylated ERK1/2, p38 and EGFR (Tyr992) by western blotting. Total ERK1/2, p38 and EGFR were used as controls.

Despite the high proportion of FPR-expressing cells in U-87MG cell line, it also contained subsets that were negative for FPR. By single-cell cloning, we isolated an F9 subclone that expressed high level of FPR at both protein and mRNA levels. In contrast, a G3 subclone was entirely negative for FPR (Figure 1A and B). Functionally, the FPR agonist fMLF induced robust chemotaxis and calcium flux responses in F9 cells, but not in G3 cells (Figure 1C). As reported (Zhou *et al*, 2005; Huang *et al*, 2007), fMLF also induced the phosphorylation of MAPKs, Akt and EGFR in F9, but not G3, cells (Figure 1D). After at least 15 passages, both F9 and G3 cells maintained their FPR-positive and -negative states, respectively.

### Phenotype of FPR^+^ and FPR^−^ subclones

**Figure 2:**
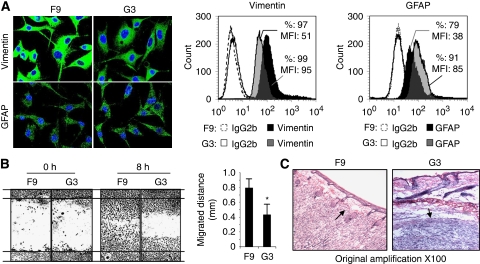
Morphology and motility of F9 and G3 cells. (**A**) Analysis of the expression of GFAP and vimentin in F9 and G3 cells. Anti-vimentin and GFAP antibodies were used to label F9 and G3 cells. The proteins were visualised (green) by an FITC-conjugated secondary antibody under confocal microscope. Nuclei were visualised in blue with DAPI. (**B**) Motility in wound-healing model. F9 and G3 cells grown to confluence on plastic were scratched to create a wound. Cells in 10% FCS/DMEM were photographed at 0 and 8 h to assess the mean distance (mm) of leading cells moving towards the ‘wound’ area. ^*^Significantly slower locomotion of G3 cells (*P*<0.01) as compared with F9 cells. (**C**) Invasiveness of xenograft tumours. F9 and G3 cells at 5 × 10^6^ cells in 100 *μ*l PBS per mouse were injected subcutaneously into the flanks of athymic mice. After 30 days, the xenograft tumours were sectioned and stained by HE. Arrows indicate F9 tumour intruding mouse dermis and well-encircled G3 tumour.

Both F9 and G3 subclones expressed vimentin, a marker for astroglial cell precursors, with lower levels in the FPR^−^ G3 cell subclone. As compared with F9 cells, G3 cells expressed a higher level of GFAP, a glial differentiation maker (Figure 2A), suggesting that although both subclones were from a GBM cell line, the FPR^+^ clone F9 was more poorly differentiated as compared with the FPR^−^ clone G3.

We next compared the mobility of FPR^+^ F9 and FPR^−^ G3 cells in a wound-healing model by creating a gap in a confluent cell monolayer. We found that the FPR^+^ F9 cells moved more rapidly than the FPR^−^ G3 cells to fill the gap in the cell monolayer (Figure 2B). In nude mice, although both subclones formed tumours, only the FPR^+^ F9 tumours invaded the surrounding mouse connective tissues with disruption of the dermis. In contrast, tumours formed by G3 cells were encapsulated by intact fibrous membrane without apparent invasion of normal mouse tissues by tumour (Figure 2C). These results indicate that in a heterogeneous human GBM cell line, the FPR^+^ cells exhibit a more motile and invasive behaviour as compared with FPR^−^ counterpart cells.

### Enhanced FPR^+^ cell motility and adhesion *in vitro*

To more precisely study the contribution of FPR to tumour cell motility and invasiveness, we transfected FPR into FPR^−^ G3 cells. Flow cytometry confirmed FPR expression in transfected G3 cells (G3F), but not in mock-transfected G3 cells (G3M) (Supplementary Figure 1A). Functionally, FPR agonist fMLF induced chemotaxis, calcium flux (Supplementary Figure 1B) and phosphorylation of Erk1/2 and p38 MAPKs as well as Akt in G3F cells (Supplementary Figure 1C). Stimulation of FPR in G3F cells also transactivated EGFR by inducing phosphorylation of the tyrosine residue 992 (Supplementary Figure 1C). Thus, transfection of FPR in an FPR^−^ subclone of GBM line enabled these cells to exhibit full agonist-induced responses as shown by tumour cells constitutively expressing FPR (Zhou *et al*, 2005; Huang *et al*, 2007).

**Figure 3:**
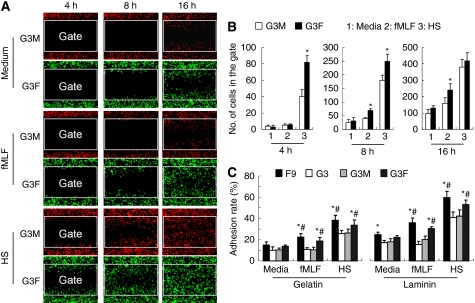
The GBM cell motility and adhesion *in vitro*. (**A**, **B**) Cell motility. The motility of CMPTX-labelled G3M (red) and CMFDA-labelled G3F (green) in wound-healing model were assayed in the presence of 100 nM fMLF or 10% HS by time-lapse photographing under confocal microscope (**A**). The cell number in a rectangle gate was counted after 4, 8 and 16 h (**B**). ^*^Indicates significant increased cell number of G3F cells as compared with G3M cells and media (*P*<0.05). (**C**) Adhesion. Serum-starved F9, G3, GM and G3F cells were cultured on 0.5% gelatin and 30 *μ*g ml^–1^ laminin-coated culture plates at 37°C for 30 min followed by washing with ice-cold PBS. The adhesion rate was the percentage of adhered cells in total input cells. ^*^Indicates significantly increased adhesion of FPR-expressing cells (F9 and G3F) as compared with G3M cells (*P*<0.05). Significantly increased cell adhesion as compared with cells treated with medium alone (Media) (P<0.05).

We then measured the motility of G3F and G3M cells in the wound-healing model *in vitro*. As shown in Figure 3A and B, G3F cells labelled with green fluorescence showed more rapid locomotion than G3M cells towards the centre of the gap created on cell monolayer in the presence of 100 nM fMLF or 10% human serum (HS). A time-lapse imaging of the migration in the ‘wound’ of G3F and G3M cells in medium with 10% HS is shown in Supplementary Movie 1. In addition, G3F and F9 cells cultured in 100 nM fMLF and 10% HS showed increased adhesion on gelatin- and laminin-coated plastic than their respective control G3M and G3 cells (Figure 3C). These results confirm that FPR expression enables GBM cells to exhibit higher level of motility and adhesion in the presence of FPR agonist fMLF or HS.

### MMP production and growth of G3F cells *in vitro*

**Figure 4:**
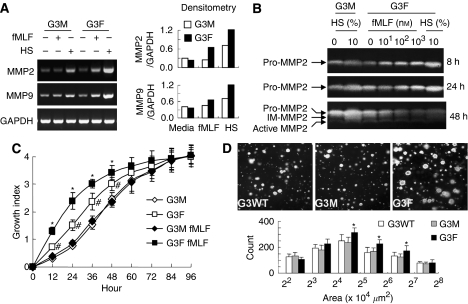
The MMP expression and cell growth *in vitro*. (**A**) Expression of MMP2 and MMP9. G3F cells were cultured in the presence or absence of fMLF or 10% HS for 24 h at 37°C, followed by RT–PCR detection of mRNA for MMP2 and MMP9. (**B**) Zymogram. G3M and G3F cells were cultured in the presence or absence of 100 nM fMLF or 10% HS for 8, 24 and 48 h at 37°C. The supernatants were collected to measure the proteolytic activity by gelatin zymography. (**C**) Growth of FPR-transfected cells in culture. The FPR-transfected G3F or mock G3M cells were cultured in 0.5% FCS/DMEM containing 10% AlamarBlue with or without 1 *μ*M fMLF. Cell growth was monitored by measuring the absorbance at 570 and 600 nm. The results are presented as the mean of ‘growth index’ (±s.e., *n*=6). ^#^Indicates significantly increased growth of G3F cells as compared with G3M cells (*P*<0.05) and ^*^indicates significantly increased growth of G3F cells in the presence of fMLF as compared with growth in the absence of fMLF (*P*<0.05). (**D**) Colony formation in soft agar. Tumour cells suspended in 200 *μ*l 0.3% agar (2500 cells) were layered on solidified bottom agar in the wells of 24-well plates. The cells were grown in DMEM containing 10% FCS at 37°C. After 3 weeks, tumour colonies were photographed under light microscopy. The results are expressed as the mean (±s.e., *n*=6) numbers of colonies. ^*^Indicates significantly increased sphere numbers formed by G3F cells as compared with G3M cells (*P*<0.05).

As increased production of MMP2 and MMP9 is associated with tumour invasion, we examined the capacity of G3F cells to produce the enzymes that are implicated in the breakdown of basement membrane by invading tumour cells. As shown in Figure 4A, the mRNA levels of MMP2 and MMP9 in G3F cells were increased when the cells were stimulated with the FPR agonist or cultured in the presence of HS. Zymography of conditioned media revealed that the levels of soluble pro-MMP-2 protein from G3F cells were higher than G3M cells (Figure 4B). In addition, fMLF increased the level of pro-MMP2 released by G3F cells. Moreover, the active MMP2 produced by G3F cells was at a higher level than that produced by G3M cells despite the absence of fMLF or HS in cell culture. We further found that G3 cells over-expressing FPR (G3F) grew more rapidly (Figure 4C) and they formed more large-size tumour colonies in soft agar (Figure 4D) as compared with mock-transfected cells G3M.

### Increased tumourigenesis, invasiveness and angiogenesis in tumours formed by G3F cells

**Figure 5:**
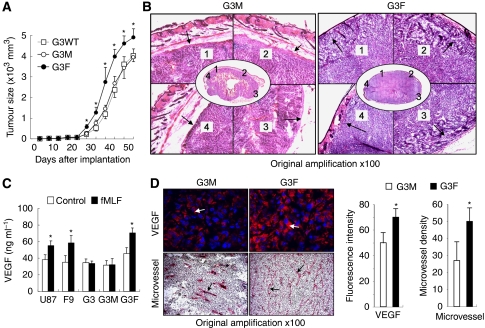
Tumourigenesis, invasiveness and angiogenesis. (**A**) Tumourigenesis in nude mice. Wild-type G3 (G3WT), G3M and G3F cells at 5 × 10^6^ cells in 100 *μ*l of PBS per mouse were injected subcutaneously into the flanks of athymic mice. Tumour size was expressed as the mean volume (in mm^3^±s.e.) of the tumours in 10 animals. ^*^Indicates significantly increased size of tumours formed by G3F cells as compared with G3M and G3WT cells (*P*<0.05). (**B**) Invasiveness *in vivo*. Sections of the xenograft tumours of G3M and G3F cells were stained by HE revealing fibrous capsule surrounding xenograft tumours. Numbers in the tumour section shown in centre panels represent areas amplified in surrounding panels. Arrows indicate tumour margin with fibrosis. (**C**) VEGF in tumour cell supernatant. The GBM cells were incubated in 24-well plates (10^4^ cells in 200 *μ*l 1% FCS DMED in each well) in the presence or absence of 100 nM fMLF for 72 h. Culture media were measured for VEGF by ELISA. ^*^Indicates significantly increased VEGF level in culture media of fMLF-containing cultures as compared with control medium (*P*<0.01). (**D**) VEGF and vessels in xenograft tumours. Frozen tumour sections having VEGF were detected by immunofluorescence staining in red. Tumour cell nuclei were revealed by DAPI in blue. The microvessels in tumours were visualised by staining of CD105. Arrows indicate VEGF fluorescence and CD105-positive microvessels. The levels of VEGF were quantified by the average optical density of the fluorescence. The microvessel densities were expressed as the mean microvessel number (±s.e.) in six high-powered fields of three independent experiments. ^*^Indicates significantly increased VEGF level and microvessel density as compared with G3M (*P*<0.05).

On the basis of *in vitro* findings that over-expression of FPR in G3 GBM subset cells transformed the cells into a more invasive and proliferative phenotype, we injected the tumour cells subcutaneously into the flanks of athymic mice and measured the rate of tumourigenesis. Tumours formed by G3F cells grew more rapidly than those formed by the wild-type or mock-transfected G3 cells (Figure 5A). Histological examination showed tumours formed by G3F cells penetrated the dermis and invaded the surrounding mouse connective tissues. In contrast, G3M tumours grew more slowly and were surrounded by intact dermis (Figure 5B).

We then measured VEGF production by U-87MG subclones *in vitro* and the density of microvessles in xenograft tumours. Stimulation of G3F, F9 cells and the parental U-87MG cells with the FPR agonist fMLF increased their production of VEGF (Figure 5C). The increased production of VEGF by FPR-expressing G3F cells *in vitro* was corroborated by significantly increased VEGF production and the microvessels in tumours formed by these cells as shown in Figure 5D. Thus, FPR expression is sufficient to promote the growth, invasion and angiogenesis of tumour cells, which were originally FPR negative and had a lesser degree of malignancy.

### Identification of potentially endogenous source of FPR agonist activity

**Figure 6:**
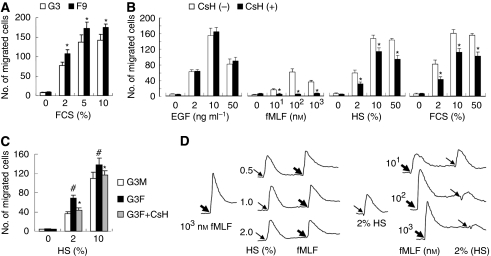
The FPR agonist activity in FCS and HS. (**A**) FCS-induced migration of F9 and G3 cells. F9 or G3 cells were measured for migratory response to 10% FCS. ^*^Indicates significantly increased migration of F9 cells in response to FCS as compared with G3 cells (*P*<0.05). (**B**, **C**) Inhibition of HS-induced tumour cell migration by FPR antagonist cyclosporin H (CsH). F9 (**B**), G3F and G3M (**C**) cells were preincubated with 10 *μ*M CsH for 30 min, then were measured for migration in response to EGF, fMLF, HS and FCS. ^*^Indicates reduced migration of CsH-treated cells as compared with medium-treated cells (*P*<0.05). ^#^Indicates increased migration of G3F cells as compared with G3M cells (*P*<0.05). (**D**) Ca^2+^ flux in G3F cells. G3F cells loaded with Fura-2 were stimulated with different concentrations of HS, followed 100 s later by 1000 nM fMLF (left panel), or the cells were stimulated with different concentration of fMLF, followed by 2% HS. The intensity of the fluorescence was recorded.

We earlier observed that the supernatants of necrotic U-87MG cells and xenograft tumour tissues of glioma cells contained FPR agonist activity (Zhou *et al*, 2005), thus may represent an endogenous source of FPR ligands in the tumour microenvironment. As FPR^+^ GBM cell clones exhibited increased motility *in vitro* in serum-containing media without the addition of exogenous FPR agonists, we examined the possibility of the presence of FPR agonists in culture media. We found that both FCS and HS were able to induce migration of GBM cell subsets *in vitro* and the FPR-expressing F9 and G3F cells showed higher levels of cell migration in response to serum-containing media as compared with the G3 and G3M counterparts, respectively (Figure 6A–C). The tumour cell migration inducing activity of FCS or HP was composed mainly of chemotactic with minor, but significant contribution of the chemokinetic element as measured by checkerboard analyses (Supplementary Table 1) in which tumour cells also migrated in the presence of negative serum gradients or equal serum concentrations in both upper and lower wells of the chemotaxis chambers. Importantly, the migration of FPR^+^ tumour cells induced by FCS or HS was partially and significantly inhibited by an FPR-specific antagonist cyclosporine H (CsH) (Wenzel-Seifert and Seifert, 1993; De Paulis *et al*, 1996), which had no effect on EGF-induced tumour cell migration (Figure 6B and C), suggesting the presence of FPR agonist(s) in FCS and HS. The HS also induced a robust intracellular Ca^2+^ mobilisation in G3F cells (Figure 6D) and attenuated the subsequent cell response to fMLF (Figure 6D). Conversely, fMLF also significantly desensitised HS-induced Ca^2+^ mobilisation in G3F cells (Figure 6D). These results indicate the presence of FPR agonists in FCS and HS and provide a basis for the increased motility of FPR^+^ tumour cells in the absence of the defined FPR agonists fMLF. Thus, tumour cells may take advantage of the presence of FPR agonists in both necrotic tumour supernatants and in normal serum to promote their growth, invasion and angiogenesis.

## Discussion

Human gliomas are heterogeneous in their degree of malignancy, ranging from more slowly growing astrocytoma to fast growing and highly invasive GBM. This study showed that even in a well-established GBM cell line, FPR is expressed at different levels by cell subsets, and an FPR-expressing subset is more motile and forms more rapidly growing invasive tumours in nude mice as compared with its FPR-negative counterpart. The FPR-expressing clone F9 also expressed higher levels of vimentin, an astroglia precursor marker, but lower levels of the differentiation marker GFAP than the FPR-negative subset G3. This is consistent with the findings with surgically removed human glioma specimens, in which FPR expression is associated with poorly differentiated tumours, and within individual human gliomas, tumour cells frequently exhibit remarkable heterogeneity (Zhou *et al*, 2005). This may explain our findings that 3 out of 14 anaplastic astrocytoma were negative for FPR and 2 out of 13 of less invasive astrocytoma are positive for FPR (Zhou *et al*, 2005). The presence of highly proliferative and invasive cell populations in a given solid tumour determines the rate of tumour progression and the lethality to the host. The U87 glioblastoma cell line contains both FPR^+^ and FPR^−^ subpopulations: although the FPR^+^ subpopulation represented by F9 clone, the G3 subclone is FPR^−^ as reported in this study. However, both the FPR^+^ F9 subclone and the FPR^−^ G3 subclone express EGF receptor and proliferate in response to EGF. Therefore, in the heterogeneous U87 cell line, the FPR^+^ cells did not show apparent growth advantage over FPR^−^ cells *in vitro*. This may explain why FPR^−^ cells may sustain their growth over longitudinal passaging and are uncoverable in the heterogeneous population. The FPR gene has a single copy in FPR^+^ U87 cells. Over a period of 15 passages, these FPR^+^ cells represented by the F9 clone maintained the same level of FPR expression. However, it is interesting to note that it has been reported that glioma stem-like cells isolated from U87 cell line and primary human gliomas express FPR (Yao *et al*, 2008b) and such stem-like cells differentiate *in vitro* to yield both FPR^+^ and FPR^−^ descendents. Therefore, it seems that FPR^+^ malignant glioma cells may represent a more poorly differentiated cell population with higher degree of malignancy.

One of the most important features of malignant tumour cells is their capacity to break the barrier of surrounding normal host tissues. This invasion process is known to depend not only on tumour cell motility, but also on tumour cell-secreted MMPs to degrade ECM. The MMPs also cleave and activate other growth factors such as TGF-*β* that are implicated in GBM motility and proliferation.

The expression of MMPs in tumour cells is induced by cytokines, growth factors, tumour promoters, physical stress, oncogenic transformation, and cell–matrix and cell–cell interactions. Several MMP genes are inducible by extracellular stimuli, which activate the AP1 transcription-factor complex through pathways involving MAPKs (ERK1/22, JNKs and p38) and PKC; PKC is also known to have a function in increasing MMP9 expression by malignant glioma cells (Yabkowitz *et al*, 1999) through cytoskeletal changes and NF-*κ*B (Chintala *et al*, 1998). Activation of FPR in myeloid and GBM cells triggers these signalling events (Zhou *et al*, 2005; Kam *et al*, 2007); therefore, provide mechanistic basis for its ability to regulate the production and activation of MMPs. The capacity to mediate MMP production may be a common feature for chemoattractant GPCRs. For instance, an FPR variant receptor, FPRL1, in monocytic cells (Lee *et al*, 2005) and a chemokine GPCR CXCR3 in colon cancer cells have also been shown to up-regulate MMP9 (Zipin-Roitman *et al*, 2007). Thus, GPCRs have an important function in increasing the proteolytic processes favouring leucocyte infiltration of tissues and tumour dissemination.

Progression of malignant tumours depends on timely neovascularisation in tumour and surrounding tissues. One of the most potent angiogenic factors produced in solid tumours is VEGF, which not only acts on vascular endothelial cells, but also increases the survival, migration and invasion of many tumour cells bearing VEGF receptors. In addition, VEGF is a potent suppressor of antigen-presenting cells in tumour microenviroment, contributing to the establishment of immune privilege of tumours. Malignant gliomas, notably GBMs, are characterised by vigorous angiogenesis and the production of copious amounts of VEGF. Our earlier study showed that stimulation of FPR in human GBM cells increased cell production of VEGF (Zhou *et al*, 2005) and another angiogenic chemokine IL8 (CXCL8) (Yao *et al*, 2008a). This study indicates that expression of FPR alone in originally FPR-negative tumour cells is sufficient for tumour cells to produce VEGF in conventional culture conditions in the presence of FCS or HS, and stimulation with FPR agonist peptide fMLF further increased VEGF production. The more vigorous angiogenesis by FPR^+^ GBM cells was further shown by higher microvessel density in tumours formed by such tumour cells, indicating a pivotal function of FPR in enhancing the angiogenic and vasculogenic process in GBM. It is interesting to note that MMPs have been reported to also act as ‘angiogenic switch’ in tumour neovascularisation (Bergers *et al*, 2000). Thus, FPR may promote GBM angiogenesis through direct induction of angiogenic factors and through elevated MMPs in tumour microenvironment.

Our study showing FPR agonist activity in the serum and supernatants of necrotic tumour cells (Zhou *et al*, 2005) strongly suggests that this receptor expressed by GBM cells may interact with host-derived agonists *in vivo*. Although the precise chemical nature of FPR agonist(s) contained in serum and necrotic tumour supernatants remains to be clarified, our preliminary results suggest that the FPR agonists are of small molecule peptide nature (Zhou *et al*, 2005). Plasma is the most complex human-derived proteome known to contain proteins derived from blood cells and other tissues including those released by normal or damaged cells and aberrant proteins from tumour cells. As proteins in the circulatory system are exposed to a variety of proteases and peptidases, plasma is rich in peptides of various size to constitute ‘peptidome’. In this context, some endogeneous agonists of FPR and its variant receptors have been identified, such as the neutrophil granule protein cathepsin G (Sun *et al*, 2004), cleaved peptide from urokinase plasminogen activator receptor (Resnati *et al*, 2002), a fragment of heme-binding proteins (F2L) (Migeotte *et al*, 2005) and formylated peptides from mitochondria (Rabiet *et al*, 2005). In addition, a phospholipid-binding protein annexin I, which was reported to be a ligand for FPR and its variant receptors, has been implicated in promoting the invasiveness of a human intestinal cancer cell line and a mouse melanoma cell line (Babbin *et al*, 2006; Rondepierre *et al*, 2009). Although the molecular nature of the FPR-stimulating activity in sera remains to be elucidated, our further studies showed that the chemotactic activity and colony-stimulating activity contained in the supernatant of necrotic GBM cells were depleted by immunoabsorption with an antibody against annexin I (Yang *et al*, manuscript in preparation), suggesting that annexin I is likely a major component responsible for the FPR-stimulating activity released by necrotic GBM cells. Thus, identification and characterisation of FPR agonist(s) in human GBM tissues, which may activate the receptor on tumour cells will shed light on the mechanisms of tumour–microenvironment interaction.

In summary, malignant tumours exploit their microenvironment to favour their survival, growth, invasion of fertile ‘soil’, and angiogenesis by ‘hijacking’ receptors involved in physiological processes to sense agonists present in the intercellular milieu. The FPR behaves as an oncoprotein that confers a highly malignant phenotype on GBM cells with increased cell motility, adhesion and release of proteases and production of angiogenic factors. Our recent study (Huang *et al*, 2008b) found that the aberrant expression of FPR in highly malignant GBM cells is closely associated with increased methylation of the p53 tumour suppressor gene promoter that reduced the capacity of p53 to repress the active transcription of FPR gene. Consequently, treatment of FPR-expressing GBM cells with methyltransferase inhibitor or transfection of the wild-type p53 reduced FPR expression and promoted GBM cell differentiation. Thus, studies of the regulation and signal transduction of FPR in GBM may yield novel molecular targets for anti-glioma therapy.
