# Supplementary material for: The G-protein-coupled formylpeptide receptor FPR confers a more invasive phenotype on human glioblastoma cells
Source: Br J Cancer. 2010 Mar 2;102(6):1052–60. doi: 10.1038/sj.bjc.6605591 (PMC2844039; doi:10.1038/sj.bjc.6605591)
Supplement: Supplementary Figure 1 and Table 1 [file 6605591x1.doc]

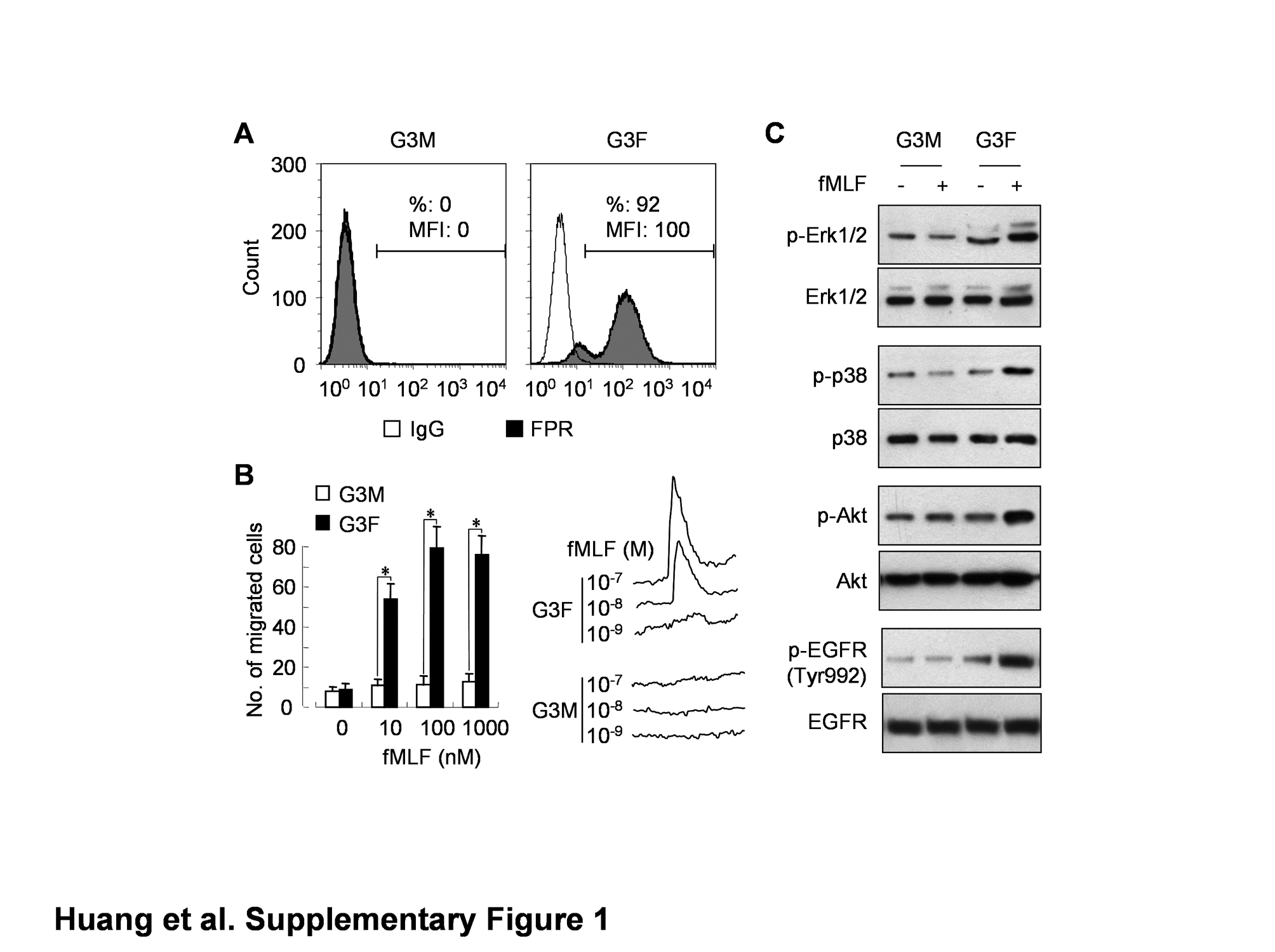


**Supplementary Figure 1. Generation of FPR^+^ G3 cells.** G3 cells were transfected with pcDNA3 plasmid containing FPR coding region (G3F) and blank pcDNA3 (G3M). **A: FACS analysis of FPR expression on G3F and G3M cells.** FPR expression was measured by a PE-conjugated mouse monoclonal antibody against FPR.%: percentage of FPR positive cells. MFI: mean fluorescence intensity. **B: fMLF-induced chemotaxis and Ca^2+^ flux.** Chemotaxis of G3F and G3M cells were assayed by 48-well chemotaxis chambers in response to different concentrations of fMLF. The results are expressed as the mean (± SE) migrated cells in 3 high powered fields of three independent experiments. * indicates significantly increased G3F migration as compared to G3M cells (p < 0.05). fMLF-induced Ca^2+^ flux was also measured in G3F and G3M cells. **C. FPR-mediated activation of signal transduction molecules.** After G3M and G3F cells were stimulated with 100 nM fMLF for 10 min, the phosphorylation of Erk2/1, p38, Akt and EGFR (residue Tyr992) were assayed by Western blot.

**Supplementary Table** 1. Checkerboard analysis of F9 GBM cell migration in the presence of HS

| HS in lower  wells (%) | HS in upper wells (%) | | | | | | | | | | | |
| --- | --- | --- | --- | --- | --- | --- | --- | --- | --- | --- | --- | --- |
|  | 0 | | | 8 | | | 16 | | | 32 | | |
| 0 | 8 | ± | 1 | 6 | ± | 2 | 5 | ± | 2 | 20 | ± | 4* |
| 8 | 61 | ± | 14* | 28 | ± | 6* | 26 | ± | 4* | 23 | ± | 11 |
| 16 | 89 | ± | 16* | 58 | ± | 11* | 40 | ± | 7* | 25 | ± | 8 |
| 32 | 89 | ± | 9*** | 80 | ± | 4*** | 52 | ± | 4*** | 32 | ± | 8* |

F9 (5 × 10^4^) cells suspended in 50 μL DMEM with 0%, 8%, 16% and 32% HS were placed in the upper wells of the chemotaxis chamber and 30 μL DMEM with 0%, 8%, 16% and 32% HS were placed in the lower wells. The wells were separated by a 10-μm (pore-size) polycarbonate filter coated with collagen type I. After incubation for 5 h, cells that migrated across the filter were counted. * indicates significantly increased number of migrated cells as compared to cell migration without HS in upper and lower wells (0) (* p < 0.05, ** p < 0.01, *** p < 0.005).

**Supplementary movie 1. Motility of G3M and G3F cells.** Equal number of CMPTX labeled G3M (Red) and CMFDA labeled G3F (Green) cells were mixed and grown in 6 cm culture dishes in DMEM containing 10% human serum. After confluence, a gap was created by scratching the center of cell monolayer. The time-lapse fluorescence images (48 images in 8 h) of the cells were collected under laser confocal microscopy. The movie was constructed by software Zeiss LSM Image Browser (Carl Zeiss GmbH).
